# Long-term outcomes of single-incision laparoscopic surgery for colorectal cancer, a single-center, open-label, randomized controlled trial

**DOI:** 10.1097/JS9.0000000000002788

**Published:** 2025-06-23

**Authors:** Zijia Song, Yiqing Shi, Xianze Chen, Yimei Jiang, You Li, Changgang Wang, Jun Li, Yaqi Zhang, Haiyan Huang, Kun Liu, Ren Zhao

**Affiliations:** Department of General Surgery, Ruijin Hospital, Shanghai Jiaotong University School of Medicine, Shanghai, China

**Keywords:** colorectal cancer, conventional laparoscopic surgery, long-term outcomes, randomized controlled trial, single-incision laparoscopic surgery

## Abstract

**Background:**

The efficacy of single-incision laparoscopic surgery (SILS) for colorectal cancer remains controversial due to uncertainties regarding long-term outcomes. This study aimed to compare the 5-year outcomes of SILS and conventional laparoscopic surgery (CLS) for colorectal cancer in a randomized clinical trial.

**Methods:**

This trial was a single-center, open-label, non-inferiority, randomized clinical trial conducted at our hospital. Patients aged 18–85 years who were diagnosed with or suspected of having colorectal cancer (staged cT1-4aN0-2M0) were enrolled. Neither the patients nor investigators were blinded to treatment allocation. The final follow-up date was on August 1, 2024. Participants were randomly assigned to either the SILS or CLS group in a 1:1 ratio using the random number table method. Both groups underwent surgery following the same oncological principles, including complete mesocolic excision for colon cancer and total mesorectal excision for rectal cancer with D3 lymph node dissection. The primary outcome was the early morbidity rate, which was reported previously. Here, we primarily report the long-term outcomes analyzed in the modified intention-to-treat (mITT) population, including 5-year disease-free survival (DFS), overall survival (OS), incisional hernia incidence, and recurrence patterns.

**Results:**

A total of 200 patients were enrolled between June 28, 2017 and June 29, 2019. Of these, 193 (110 men [57.0%]; median (interquartile range [IQR]) age, 64 [15] years) were included in the mITT analysis. No patients were lost to follow-up within 5 years postoperatively. The median follow-up was 71.3 months (IQR 64.2–76.8). The 5-year DFS was 86.6% in the SILS group and 86.5% in the CLS group (hazard ratio, 1.03 [95% CI 0.47–2.21], *P* = 0.95). The 5-year OS rates were 88.7% and 90.6% in the SILS and CLS groups, respectively (hazard ratio, 1.26 [95% CI 0.52–3.02], *P* = 0.61). No statistically significant differences were observed between the two groups in terms of recurrence patterns, incisional hernia incidence, or recurrence and survival rates stratified by tumor stage.

**Conclusions:**

SILS, when performed by experienced surgeons, can be considered a promising alternative for selected colorectal cancer patients. This potentially expands the range of surgical treatment options available to both patients with colorectal cancer and surgeons.

## Introduction

Single-incision laparoscopic surgery (SILS) is a novel surgical technique derived from conventional laparoscopic surgery (CLS), in which multiple laparoscopic instruments are placed through a single small abdominal incision to perform the operation. Since its introduction, SILS has attracted considerable interest as a means to further reduce the invasiveness of surgical procedures, thereby establishing itself as a highly desirable approach in a range of surgical fields, including gynecological, urological, and general surgery.

The performance of SILS is more technically challenging than that of CLS because of the loss of triangulation, in-line orientation, and instrument collision. These factors complicate the execution of precise movements necessary for complex procedures, such as radical colorectal surgery, which requires multiple surgical field changes, thorough lymph node dissection, and tension-free anastomosis. The technical demands of SILS have led to a slower adoption curve, with the first reported single-incision laparoscopic radical left hemicolectomy by Bucher *et al*[1] was not described until 2009.

Despite these challenges, SILS is currently viewed as a significant advancement in minimally invasive colorectal surgery[2]. Potential benefits include further reduced surgical trauma, less postoperative pain, better cosmetic outcomes, lower medical costs, and more efficient use of human resources. However, despite the demonstration of the short-term safety and feasibility of SILS in several retrospective studies^[3–9]^, meta-analyses^[10–14]^, and randomized controlled trials (RCTs)^[15–24]^, its use remains limited. This limitation is partly due to the challenging nature of the surgical procedure and limited availability of the requisite equipment. Moreover, the lack of comprehensive long-term outcome data on SILS for colorectal cancer, particularly in the context of RCTs, raises concerns regarding the oncological prognosis of this technique and the potential for an increased incidence of incisional hernia.

Further studies, particularly RCTs that focus on long-term outcomes, are required to ascertain the efficacy of SILS for colorectal cancer. The short-term outcomes of this RCT have been previously reported[24]. The objective of this study was to present the long-term outcomes, including 5-year disease-free survival (DFS) rates, overall survival (OS) rates, incidence of incisional hernia, and recurrence patterns. In compliance with the Transparency In The Reporting of Artificial INtelligence–the TITAN guideline[25], we confirm that no artificial intelligence (AI) tools were used at any stage of this study or in the preparation of the manuscript.

## Methods

### Design

This single-center, open-label, randomized non-inferiority trial was conducted at the Department of General Surgery in our hospital. The trial was approved by the Clinical Trial Ethics Committee of our hospital. The study protocol has been described in detail in a previously published article[24]. This trial was registered at ClinicalTrials.gov. The work was reported in line with Consolidated Standards of Reporting Trials (CONSORT) criteria[26].

### Participants

Patients aged 18–85 years with a confirmed or suspected diagnosis of colorectal cancer were screened for eligibility. The inclusion criteria were as follows: (1) 18 years < age < 85 years; (2) pathological or highly suspected colorectal carcinoma; (3) tumor located in colon and rectum (the lower border of the tumor is above the anterior peritoneal reflection); (4) clinically diagnosed cT_1-4a_N_0-2_M_0_ lesions according to the 7th Edition of American Joint Committee on Cancer Staging Manual; (5) tumor size of ≤5 cm; (6) performance status Eastern Cooperative Oncology Group (ECOG) 0-1; (7) The American Society of Anesthesiologists class I–III; (8) informed consent.

The exclusion criteria were as follows: (1) Body mass index (BMI) >30 kg/m^2^; (2) the lower border of the tumor is located distal to the anterior peritoneal reflection; (3) previous gastrointestinal surgery (except appendectomy); (4) emergency operation due to complication caused by colorectal cancer (bleeding, perforation or obstruction); (5) requirement of simultaneous surgery for other disease; (6) pregnancy or lactation; (7) severe mental disease; (8) simultaneous or metachronous multiple cancers with DFS i5 years.

**HIGHLIGHTS**

- The 5-year disease-free survival was 86.6% in the single-incision laparoscopic surgery (SILS) group and 86.5% in the CLS group (hazard ratio 1.03 [95% CI 0.47–2.21], *P* = 0.95). The 5-year overall survival was 88.7% in the SILS group and 90.6% in the CLS group (hazard ratio 1.26 [95% CI 0.52–3.02], *P* = 0.61).
- Subgroup analysis revealed no statistically significant differences in recurrence patterns, incidence of incisional hernia, and recurrence and survival rates by tumor stage between the groups.
- The findings support single-incision laparoscopic surgery as a viable option for selected patients with colorectal cancer in terms of long-term outcomes, expanding the range of surgical treatment options available to both colorectal cancer patients and surgeons.

Patient sex (male or female) was also self-reported. Patients were recruited via outpatient clinics, and their eligibility was confirmed by a multidisciplinary team that included surgeons, oncologists, and pathologists. Written informed consent was obtained from all patients prior to enrollment.

### Randomization and masking

Participants were randomly allocated to the SILS or CLS group in a 1:1 ratio using the random number table method. An independent data inspector, who was not involved in the patient screening and enrollment process, performed the randomization. The allocation sequence was concealed from the surgeons until the patients were formally allocated to their respective groups, using sequentially numbered, identical, opaque, sealed envelopes. Neither patients nor investigators were blinded to the treatment allocation.

### Surgical procedures

The surgical team consisted of six qualified surgeons, each with experience in over 50 CLS procedures. One senior surgeon in the team was responsible for all SILS procedures. Prior to this study, he had performed over 100 SILS and 1000 CLS procedures.

In both groups, all procedures were conducted in accordance with the same oncological principles, including complete mesocolic excision for colon cancer and total mesorectal excision (TME) for rectal cancer with D3 lymph node dissection. The TME procedure performed in this study adhered to the principles of tumor-specific mesorectal excision, representing an extended interpretation of conventional TME. Specifically, the following principles were met: (1) sharp dissection in the holy plane under direct visualization; (2) complete preservation of the mesorectal integrity; and (3) resection of the mesorectum extending 4–5 cm distal to the tumor margin.

Intestinal segment mobilization, lymph node dissection, and vascular ligation were performed laparoscopically. Specimen excision and anastomosis were performed in an open state using a small auxiliary incision. In the SILS group, all operations were performed through a trans-umbilical single incision using the SILSTM Port (Covidien, Mansfield, MA, USA) with three 5-mm cannulas inserted or the Star-Port (Surgaid®, Guangzhou, China), which consists of three fixed instrument channels (one 5-mm, two 10-mm and one 12-mm). A draining tube was placed through a transumbilical incision. In the CLS group, the operation was performed using 3–5 trocars. Conversion was defined as the use of additional trocars or laparotomy. Surgical procedures and perioperative management have been reported previously^[8,9,24]^.

### Outcomes

The primary outcome was early morbidity defined as the postoperative complications observed within 30 days of surgery. The secondary outcomes included intraoperative outcomes, postoperative pain scores, postoperative recovery, pathological outcomes, and long-term outcomes, including 5-year DFS rates, OS rates, incidence of incisional hernia, and recurrence patterns. DFS was defined as the time from the day of surgery to the date of tumor recurrence (local, regional, or distant), all-cause death, or the last follow-up, whichever occurred first. The OS rate was defined as the time from the day of surgery to the date of all-cause death or the last follow-up, whichever occurred first. Recurrence was confirmed by radiological and histological methods. An incision hernia was confirmed by physical examination or radiological methods.

Each participant was followed up at 1 and 3 months after surgery, then every 3 months for the first 2 years, and every 6 months for the next 3 years. During follow-up, postoperative adjuvant treatment, late postoperative complications, tumor recurrence and metastasis, incisional hernia, and death were recorded. When participants failed to return for follow-up visits, they were contacted via phone or email to complete the follow-up data. The follow-up schedule was defined according to the protocol.

### Statistical analysis

Sample size estimation was conducted using the PASS software (11th edition, NCSS, LLC, Utah, USA). The primary endpoint was the incidence of early morbidities. Based on data previously collected at our institution, the corresponding rates in the CLS and SILS groups were 14% and 10%, respectively. The required sample size was calculated using a one-sided alpha of 0.025, power of 0.8, and non-inferiority margin of 10%. Considering an anticipated attrition rate of 15%–20%, the sample size was increased to 200 individuals (100 per group).

Statistical analysis was conducted using the statistical software packages SPSS (version 22.0; IBM Corp., Armonk, NY, USA) and R version 4.3.2. Data were analyzed according to the modified intention-to-treat (mITT) principle. Both 5-year DFS and 5-year OS were analyzed in the per-protocol (PP) population to assess robustness of the findings. For categorical variables, the χ^2^ test or Fisher exact test was used. For continuous variables, Student’s *t*-test or Mann–Whitney *U*-test was applied. DFS and OS were estimated using the Kaplan–Meier method and compared using the log-rank test. Subgroup analysis for DFS was conducted using the Cox proportional hazards model. Statistical significance was set at *P* < 0.05.

## Results

### Study population

**Figure 1.**
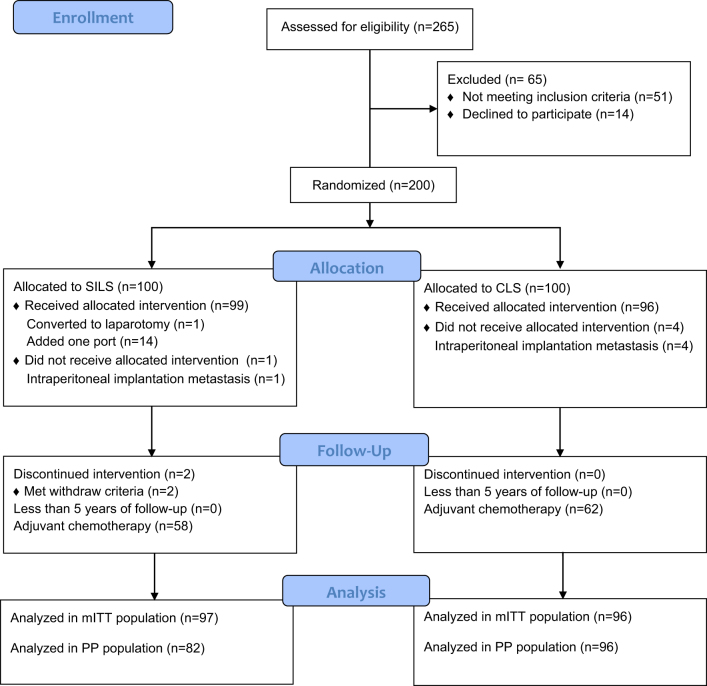
Consort flow diagram.

Between June 28 2017 and June 29, 2019, 200 patients diagnosed with or suspected of having colorectal cancer were randomly assigned to either the SILS or CLS group. In the SILS group, one patient was excluded due to the discovery of intraperitoneal implantation metastasis and two patients were found to have benign pathological lesions after surgery. In the CLS group, four patients were excluded because of the discovery of intraperitoneal implantation metastasis. Fifteen patients in the SILS group underwent surgical conversion (one converted to laparotomy, 14 added to one port) and were retained in mITT analysis but excluded from PP analysis. All patients completed 5-year follow-up without attrition. Therefore, 193 patients (97 and 96 in the SILS and CLS groups, respectively) were included in the mITT analysis and 179 patients (82 and 96 in the SILS and CLS groups, respectively) were included in the PP analysis (Fig. 1).

Tables 1 and 2 provide an overview of the baseline clinical and pathological characteristics of 193 patients, indicating no significant differences between the two groups.

**Table 1:** Baseline characteristics

Characteristics	SILS (*n* = 97)	CLS (*n* = 96)
Age, median (IQR), years	63 (54.5–69)	65 (56–70)
Sex		
Male	56 (57.7)	54 (56.3)
Female	41 (42.3)	42 (43.8)
BMI, mean (SD), kg/m^2^	23.0 (2.8)	23.6 (3.2)
ASA grade		
I	40 (41.2)	29 (30.2)
II	47 (48.5)	53 (55.2)
III	10 (10.3)	14 (14.6)
Comorbidities	51 (52.6)	53 (55.2)
Previous abdominal surgery	23 (23.7)	26 (27.1)
ECOG score		
0	43 (44.3)	36 (37.5)
1	54 (55.7)	60 (62.5)
Procedure performed		
Right hemicolectomy	23 (23.7)	26 (27.1)
Left hemicolectomy	6 (6.2)	9 (9.4)
Transverse colectomy	0 (0)	1 (1)
Sigmoidectomy	31 (32.0)	24 (25.0)
Anterior resection	37 (38.1)	36 (37.5)
Operation time, median (IQR), min	120 (90–132)	120 (96.3–148.3)
Adjuvant chemotherapy	58 (59.8)	62 (64.6)
Capecitabine	24 (24.7)	19 (19.8)
Capeox	34 (35.1)	43 (44.8)

Data are *n* (%) unless otherwise specified.

ASA, The American Society of Anesthesiologists; BMI, body mass index; ECOG, Eastern Cooperative Oncology Group.

**Table 2:** Pathologic outcomes

Variable	SILS (*n* = 97)	CLS (*n* = 96)	*P*^c^
Tumor size (cm)	3.5 (2.5–4)	4 (3–4.5)	0.071^b^
Proximal resection margins (cm)	6 (4–9)	6 (4.1–9.9)	0.422^b^
Distal resection margins (cm)	4 (2.8–7)	4.4 (2.5–7.9)	0.527^b^
Harvested lymph nodes	13 (10–15)	13 (10.2–15)	0.952^b^
Cell type			0.195^c^
WD/MD	50 (51.5)	40 (41.7)	
PD/Others	47 (48.5)	56 (58.3)	
Perineural invasion	21 (21.6)	18 (18.8)	0.720^c^
Vascular invasion	32 (33.0)	25 (26)	0.344^c^
Positive circumferential resection margin^a^	0 (0)	0 (0)	–
pT stage			0.504^c^
Tis/T1	18 (18.6)	12 (12.5)	
T2	20 (20.6)	20 (20.8)	
T3	32 (33.0)	29 (30.2)	
T4a	27 (27.8)	35 (36.5)	
pN stage			0.619^c^
N0	61 (62.9)	65 (67.7)	
N1	28 (28.9)	22 (22.9)	
N1a	11 (11.3)	10 (10.4)	
N1b	13 (13.4)	10 (10.4)	
N1c	4 (4.1)	2 (2.1)	
N2	8 (8.2)	9 (9.4)	
N2a	6 (6.2)	8 (8.3)	
N2b	2 (2.1)	1 (1)	
pTNM stage			0.671^c^
0	4 (4.1)	5 (5.2)	
I	28 (28.9)	24 (25.0)	
II	29 (29.9)	36 (37.5)	
IIA	17 (17.5)	18 (18.8)	
IIB	12 (12.4)	18 (18.8)	
III	36 (37.1)	31 (32.3)	
IIIA	6 (6.2)	1 (1)	
IIIB	25 (25.8)	26 (27.1)	
IIIC	5 (5.2)	4 (4.2)	

Data are *n* (%) or median (IQR).

MD, moderately differentiated; PD, poorly differentiated; WD, well differentiated.

^a^ Assessed in rectal cancer.

^b^ Mann–Whitney *U* test.

^c^ Fisher’s exact test.

### Disease-free survival

**Figure 2.**
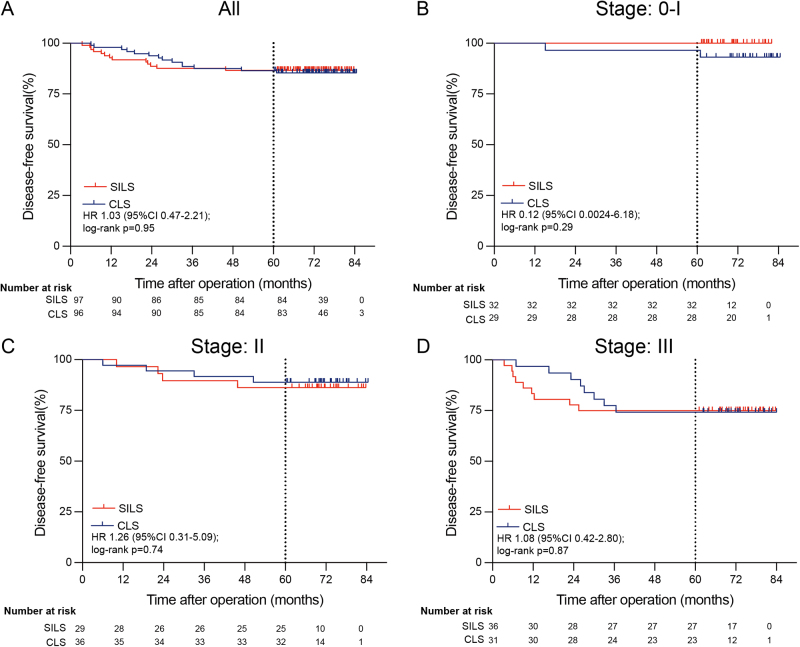
Disease-free survival for single-incision laparoscopic surgery (SILS) vs conventional laparoscopic surgery (CLS) for colorectal cancer at 5 years after surgery (mITT analysis).

The median follow-up period was 71.3 months (interquartile range [IQR] 64.2–76.8). In total, 23 patients (11.9%) experienced tumor recurrence or metastasis during the follow-up period (11 in the SILS group and 12 in the CLS group) (Table 3). All recurrences occurred within 5 years of surgery. In both groups, the liver was the most common site of recurrent metastases, with seven and six patients in the SILS and CLS groups, respectively, developing liver metastases. Two patients in each group presented with multisite metastases at the time of initial diagnosis of recurrence. In the SILS group, one patient had local recurrence and lung metastases, and another had local recurrence with liver and lung metastases. In the CLS group, two patients had liver and lung metastases. In the mITT analysis, the 5-year DFS rate was 86.6% in the SILS group and 86.5% in the CLS group (hazard ratio 1.03 [95% CI 0.47–2.21]), showing no significant difference (*P* = 0.95). For stage-specific analysis, the 5-year DFS in the SILS versus CLS group was 100% versus 96.6% for patients with stage 0-I tumors (hazard ratio 0.12 [95% CI 0.0024–6.18], *P* = 0.29); 86.2% versus 88.9% for stage II tumors (hazard ratio 1.26 [95% CI 0.31–5.09], *P* = 0.74), and 75.0% versus 74.2% for stage III tumors (hazard ratio 1.08 [95% CI 0.42–2.80], *P* = 0.87) (Fig. 2). Similarly, no significant difference was observed in the PP analysis (Supplemental Digital Content Figure 1, available at: http://links.lww.com/JS9/E422).

**Table 3:** Frequencies of causes of first recurrence and death during follow-up

Variable	SILS (*n* = 97)	CLS (*n* = 96)	*P*
Recurrence	11 (11.3)	12 (12.5)	0.83^a^
Local	0 (0)	2 (2.1)	
Liver	6 (6.2)	4 (4.2)	
Lung	1 (1.0)	3 (3.1)	
Peritoneum	2 (2.1)	1 (1.0)	
Multiple sites	2 (2.1)	2 (2.1)	
Death	11 (11.3)	11 (11.5)	1.00^a^
Colorectal cancer	9 (9.3)	9 (9.4)	
Other causes	2 (2.1)	2 (2.1)^b^	

Data are *n* (%).

^a^ Fisher’s exact test.

^b^ One patient died after 5 years postoperatively.

### Overall survival

**Figure 3.**
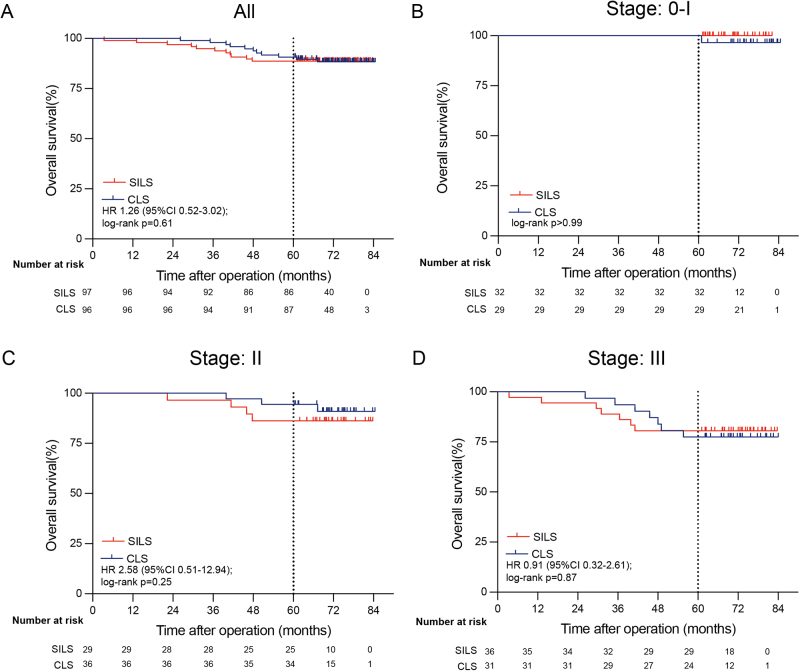
Overall survival for single-incision laparoscopic surgery (SILS) vs conventional laparoscopic surgery (CLS) for colorectal cancer at 5 years after surgery (mITT analysis).

In total, 22 patients (11.4%) died during follow-up (11 in the SILS group and 11 in the CLS group) (Table 3), with 18 (9.3%) dying due to disease progression (nine in each group) and four (2.1%) due to other causes (two in each group). Among the non-cancer-related deaths, the CLS group included one case of undetermined cause and one event occurring more than 5 years postoperatively. In the SILS group, two patients died due to cardiovascular and cerebrovascular events. The 5-year OS rate was 88.7% in the SILS group and 90.6% in the CLS group (hazard ratio, 1.26 [95% CI 0.52–3.02], *P* = 0.61). For stage-specific analysis, the 5-year OS in the SILS versus CLS group was 100% versus 100% for stage 0-I tumors (*P* > 0.99); 86.2% versus 94.4% for stage II tumors (hazard ratio 2.58 [95% CI 0.51–12.94], *P* = 0.25); and 80.6% versus 77.4% for stage III tumors (hazard ratio 0.91 [95% CI 0.32–2.61], *P* = 0.87) (Fig. 3). Similarly, no significant difference was observed in the PP analysis (Supplemental Digital Content Figure 2, available at: http://links.lww.com/JS9/E422).

### Incisional infection and incisional hernia

During postoperative follow-up, incisional infections developed in 4 (4.1%) SILS patients and 2 (2.1%) CLS patients, all of which were successfully managed with conservative wound care without subsequent hernia formation. Two cases of incisional hernia were documented during the study period: one in the SILS group at 6 months postoperatively and one in the CLS group at 20 months postoperatively. No statistically significant differences were observed between groups in either surgical site infection rates (4.1% vs 2.1%, *P* = 0.44) or incisional hernia (1.0% vs 1.0%, *P* = 1.00).

### Subgroup analysis

**Figure 4.**
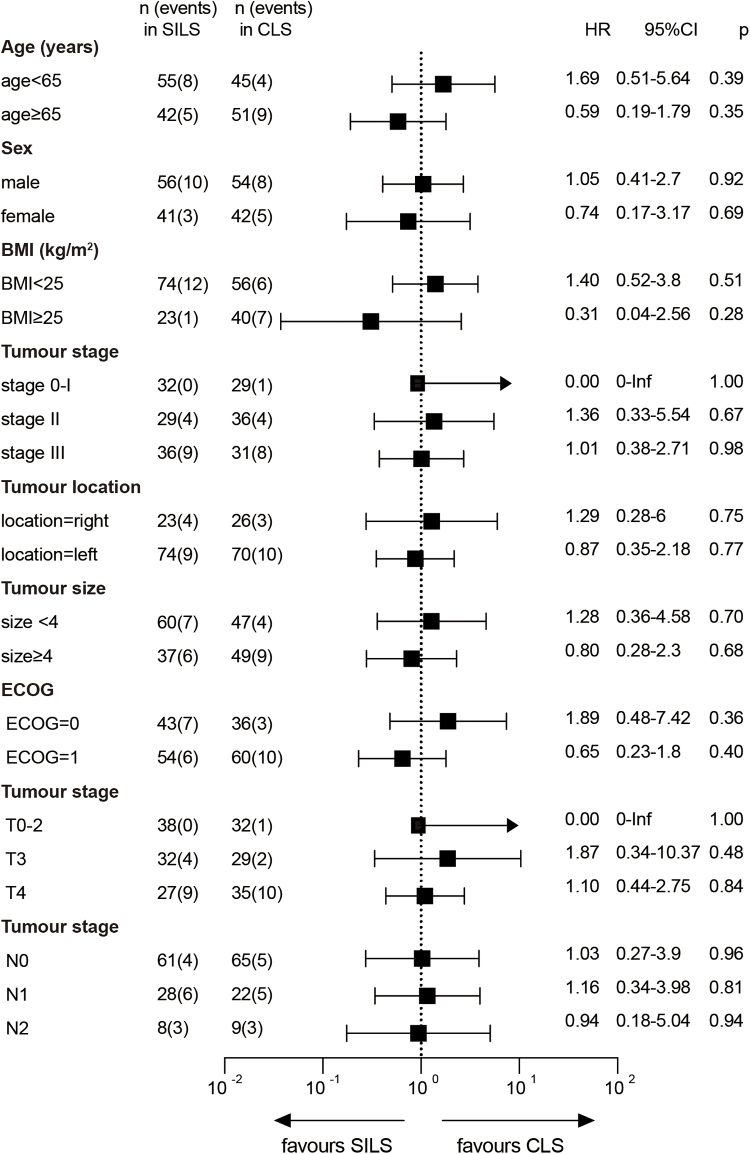
Subgroup analysis of disease-free survival (DFS).

Subgroup analyses for 5-year DFS were conducted according to age, sex, BMI, tumor stage, tumor location, tumor size, and ECOG performance status. None of the results were statistically significant (Fig. 4). Compared with CLS, SILS showed a slight numerical improvement in the subgroup of patients with a BMI ≥25, but this difference was not significant.

## Discussion

The long-term outcomes of this trial demonstrated that colorectal cancer patients who underwent SILS exhibited comparable 5-year DFS, 5-year OS, recurrence patterns, and incidence rates of incisional hernia to those who underwent CLS. There is a paucity of data regarding the long-term outcomes of SILS for colorectal cancer. To the best of our knowledge, only two retrospective studies^[27,28]^ have reported 5-year long-term outcomes, and only one of the nine previously published RCTs of SILS for colorectal cancer has reported long-term outcomes[23]. In the aforementioned RCT report, the 5-year DFS rates were 91% and 88% in the SILS and CLS groups, respectively, and the 5-year OS rates were 95% and 93%, respectively, with no statistically significant differences. The observed rates were slightly higher than those reported in our study, potentially due to the inclusion of a greater number of patients with stage II (SILS, 29 [29.9%]; CLS, 36 [37.5%]) and stage III (SILS: 36 [37.1%], CLS: 31 [32.3%]) disease in our study. In contrast, Watanabe et al.’s study[23] included 16 (16%) stage II patients and 28 (28%) stage III patients in the SILS group and 25 (25%) stage II and 21 (21%) stage III patients in the CLS group. Additionally, patients with lesions in the transverse colon, descending colon, or rectum were excluded from the study by Watanabe et al. Conversely, rectal cancer patients with tumor lower margins located above the anterior peritoneal reflection accounted for a significant proportion of our study population (SILS group: 37 [38.1%]; CLS group: 36 [37.5%]). In these populations, the 5-year DFS was 81.1% in the SILS group and 80.6% in the CLS group (hazard ratio 1.05 [95% CI 0.37–3.00], *P* = 0.92), whereas the 5-year OS was 83.8% in the SILS group and 86.1% in the CLS group (hazard ratio, 1.23 [95% CI 0.38–4.02]; *P* = 0.73). These findings are comparable to those of a previous case-matched study[28], which was the only study to report 5-year results of using SILS to treat mid-high rectal cancer. Thus, our findings add to the evidence for the oncological safety of SILS in the treatment of mid-high rectal cancer.

Similar to previous studies^[23,27–29]^, the pattern of recurrent metastasis in the SILS group was not specific in the present study, with liver metastasis being the most common site of metastasis. No implantation metastases were observed from the incision.

A trend towards worse survival in the SILS group was observed for patients with a BMI >25 kg/m^2^ and clinical stage III disease in Watanabe *et al*’s study[23], possibly due to the technical challenges of SILS. However, this trend was not found in the present study; instead, SILS showed a slight numerical improvement in the subgroup of patients with BMI ≥25 kg/m^2^ in the present study. This may be attributed to the different levels of experience of the surgeons. In this study, a single senior surgeon was responsible for all SILS operations. He had extensive experience in SILS and CLS, while the CLS group consisted of six surgeons with varying levels of experience, potentially introducing bias in the quality of surgery.

While previous studies^[30–32]^ have suggested a higher incidence of incisional hernias with SILS, this study observed only one case of incisional hernia in the SILS group, despite the placement of a drainage tube through the incision. Similarly, in the case-matched study conducted by Yun *et al*[29], 239 patients who underwent surgery for colorectal cancer with the SILS technique had a median follow-up period of 33 months, during which no incisional hernia was identified. These findings indicate that SILS does not markedly elevate the risk of incisional hernia formation. It seems probable that the quality of the incisional suture and the degree of postoperative incisional tension exert a more significant influence. It is noteworthy that previous studies have demonstrated that SILS is effective in reducing postoperative pain, which may contribute to a reduction in incisional tension. Furthermore, obesity is another factor associated with incisional hernias. However, this study excluded patients with a BMI >30 kg/m^2^, which warrants further research on the incidence of incisional hernias in obese colorectal cancer patients undergoing SILS.

Due to the distinct technical characteristics, SILS can hardly gain experience from CLS[33]. Haas *et al*[34] suggested that prior experience in three-port laparoscopic surgery may facilitate the transition to SILS, as both approaches share some similar technical challenges, particularly the absence of an assistant for optimal surgical field exposure. However, SILS further complicates the procedure by consolidating all three trocars into a single incision. Their study indicated that approximately 30–36 cases are required to overcome the learning curve for SILS in right colon cancer resections. Similarly, Kirk *et al*[35] reported a learning curve of 40 cases for single-incision laparoscopic right hemicolectomy, whereas Kim *et al*[36] observed a longer adaptation period of 61–65 cases for single-incision laparoscopic radical sigmoid colectomy. Based on our clinical experience, proficiency in SILS requires several key technical adjustments: (1) external hand crossing and internal instrument crossing to restore triangulation, (2) strategic patient positioning and suspension techniques to optimize exposure, and (3) close coordination between the surgeon and the scopist to minimize instrument collisions.

The advent of revolutionary innovations and advances in surgical procedures has been shaped by a history of persistent challenges. For instance, laparoscopic surgery, which was initially met with skepticism as a mere “boy’s toy” over three decades ago, has since become a foundational medical technology. Single-incision laparoscopic colorectal cancer surgery represents a novel minimally invasive technology that has transformed the operational paradigm of traditional laparoscopic surgery. In contrast to the conventional approach of complete exposure followed by extensive free dissection, single-incision laparoscopic colorectal cancer surgery is a gradual process of incremental local freedoms, which enables the dissection of lines and surfaces and ultimately achieves complete clearance and freedom. The foundation for performing single-incision robotic surgery and pure natural orifice translumenal endoscopic surgery (NOTES) is based on the accumulation of experience in SILS surgery. Following extensive experience in a multitude of SILS procedures, our team has subsequently demonstrated proficiency in single-incision robotic surgery[37] and pure NOTES surgery[38]. As robotic single-incision surgery can further overcome the technical difficulties of SILS surgery, it may become one of the main surgical modalities in the future, and SILS surgery has an important transitional significance.

### Limitations

This study had several limitations. First, the strict inclusion and exclusion criteria, including the exclusion of patients with BMI >30 kg/m^2^, may limit the generalizability of the findings to Western populations. Second, the concentration of SILS cases with a single experienced surgeon may introduce a potential confounding factor, particularly in operation time measurements. Third, the study design did not account for the individual learning curves of participating surgeons. As the senior surgeon had already surpassed the learning curve, the achieved optimal efficiency may not reflect outcomes during initial SILS adoption by less experienced surgeons. Additionally, the sample size of the study was based on the perioperative complication rate, potentially affecting the statistical validity of long-term outcomes. Consequently, we designed a large multicenter RCT study[39] with 3-year DFS as the endpoint to investigate the long-term outcomes of SILS.

## Conclusions

In conclusion, SILS, when performed by experienced surgeons, can be considered a promising alternative for selected colorectal cancer patients. This potentially expands the range of surgical treatment options available to both patients with colorectal cancer and surgeons.

## Data Availability

Not applicable.

## Research registration unique identifying number (UIN)

ClinicalTrials.gov Identifier: NCT03151733.

## Assistance with the study

The authors thank Dr. Wang Bingshun (Department of Biostatistics, Clinical Research Institute, Shanghai Jiao Tong University School of Medicine, 227 Chong Qing Nan Rd, Shanghai 200025, China) for help with statistical analysis of the data.
